# Obesity Triggers Enhanced MDSC Accumulation in Murine Renal Tumors via Elevated Local Production of CCL2

**DOI:** 10.1371/journal.pone.0118784

**Published:** 2015-03-13

**Authors:** Malika Hale, Farah Itani, Claire M. Buchta, Gal Wald, Megan Bing, Lyse A. Norian

**Affiliations:** 1 Department of Urology, The University of Iowa, Iowa City, Iowa, United States of America; 2 Interdisciplinary Graduate Program in Immunology, The University of Iowa, Iowa City, Iowa, United States of America; 3 Holden Comprehensive Cancer Center, The University of Iowa, Iowa City, Iowa, United States of America; 4 Fraternal Order of Eagles Diabetes Research Center, The University of Iowa, Iowa City, Iowa, United States of America; Van Andel Institute, UNITED STATES

## Abstract

Obesity is one of the leading risk factors for developing renal cell carcinoma, an immunogenic tumor that is treated clinically with immunostimulatory therapies. Currently, however, the mechanisms linking obesity with renal cancer incidence are unclear. Using a model of diet-induced obesity, we found that obese BALB/c mice with orthotopic renal tumors had increased total frequencies of myeloid-derived suppressor cells (MDSC) in renal tumors and spleens by d14 post-tumor challenge, relative to lean counterparts. Renal tumors from obese mice had elevated concentrations of the known myeloid cell chemoattractant CCL2, which was produced locally by increased percentages of dendritic cells, macrophages, B cells, and CD45^-^ cells in tumors. MDSC expression of the CCL2 receptor, CCR2, was unaltered by obesity but greater percentages of CCR2^+^ MDSCs were present in renal tumors from obese mice. Of note, the intracellular arginase levels and per-cell suppressive capacities of tumor-infiltrating and splenic MDSCs were unchanged in obese mice relative to lean controls. Thus, our findings suggest that obesity promotes renal tumor progression via development of a robust immunosuppressive environment that is characterized by heightened local and systemic MDSC prevalence. Targeted intervention of the CCL2/CCR2 pathway may facilitate immune-mediated renal tumor clearance in the obese.

## Introduction

In the United States nearly 35% of adults are obese, and nearly 20% of all cancers are considered to be obesity-linked [1,2]. Obese individuals (Body Mass Index or “BMI” >30) have a 50–75% increased risk for developing renal cell carcinoma (RCC), making obesity one of the leading risk factors for developing this type of cancer [3,4]. In addition, several studies have determined that obese individuals with RCC have a worse prognosis and heightened mortality rates relative to lean individuals, although other reports have produced contradictory findings [5–8].

Despite the documented connections between obesity and increased RCC risk, the mechanisms underlying these relationships remain unclear. One potential link is that of obesity-associated chronic inflammation as a promoter of tumor progression. With the onset of obesity, adipocytes and adipose-tissue macrophages produce a variety of pro-inflammatory cytokines, including IL-6, IL-1β and TNFα [9,10]. The concentrations of these cytokines increase as obesity develops, reflecting changes in adipocyte biology and altered leukocyte composition in adipose tissues. Chronic inflammation has been implicated in the development of multiple obesity-associated pathologies, including the elevated rates of tumorigenesis seen in obese mice [11].

Even in the absence of obesity, solid tumors are characterized by chronic inflammation, and this prolonged inflammatory state has been shown to diminish protective immunity [12]. One mechanism of inflammation-mediated tumor suppression is the accumulation of Myeloid-Derived Suppressor Cells (MDSCs). MDSCs are immature, myeloid-lineage cells that are recruited to solid tumors by local production of pro-inflammatory cytokines and chemokines [13–15]. Numerous studies have shown that MDSC are key contributors to tumor-associated immune suppression, and we and others have identified them in the peripheral blood and tumors of human subjects with RCC [16–18].

Connections between obesity, tumor growth, and anti-tumor immunity are poorly understood, and the effects of obesity on MDSC accumulation and function in tumor-bearing mice have not been thoroughly studied. One prior report established that tumor-free, obese mice accumulate cells with an MDSC phenotype, although the suppressive capacity of these cells was not examined [19], and a second report found that obese mice accumulated MDSC in renal tumors but did not examine potential causes of MDSC accumulation [20]. We found previously that obese mice had equivalent outgrowth of renal tumors compared to lean mice, but had decreased responses to immunotherapy, resulting in fatal renal tumor outgrowth [21]. Decreased immunotherapeutic efficacy in obese mice was associated with a variety of dendritic cell and CD8 T cell defects [21]. Here, we used the same model of diet-induced obesity (DIO) and orthotopic renal tumor challenge to explore the hypothesis that concomitant tumor growth and obesity promote chemokine changes in the local tumor environment that lead to increased MDSC accumulation relative to what is seen in lean tumor-bearing animals. Our results provide an improved understanding of the immune environment that arises when obesity accompanies tumor growth and further contribute to our understanding of immunotherapeutic failure in obese animals. Ultimately, our findings may be informative in the development of effective immunotherapies for obese cancer patients, as they suggest that MDSC-mediated immune suppression may be more pronounced in these individuals.

## Materials and Methods

### Animals and Ethics Statement

Female BALB/c mice were purchased (NCI) at 7–8 weeks of age, maintained on standard chow for one week after receipt, then randomly assigned to either standard chow or high fat diet (HFD) (Research Diets # 12492, 60% kcal from fat) for 20 weeks. Mice were housed 5 to a cage under pathogen-free conditions at the University of Iowa Animal Care Facility, a fully accredited Association for Accreditation of Laboratory Animal Care facility. All animal procedures were approved by the University of Iowa Institutional Animal Care and Use Committee (License # A3021–01), and were carried out in strict accordance with recommendations contained within the Guide for the Care and Use of Laboratory Animals of the National Institutes of Health. After 20 weeks on feed, all mice were weighed, and the mean and s.d. of the standard chow or “normal weight” (NW) group were calculated. Mice in the HFD group were defined as being diet-induced obese (DIO) if their body weight was >3 s.d. above the NW group mean [21]. Representative weights for DIO and lean/NW BALB/c mice in our colony were previously reported [21].

### Tumor cell line and tumor challenge

Culture and use of the murine renal adenocarcinoma cell line Renca has been described [22,23]. Intra-renal (IR) tumor challenge was performed as described [22]; some mice in each experiment remained as tumor-free controls. Prior studies had established that inflammation due to the surgical procedure itself had negligible effects on ensuing immune responses [21]. Animal surgery was performed under ketamine/xylazine anesthesia, and all efforts were made to reduce animal suffering during and after surgery. Intra-renal tumor challenge in this model gives rise to renal tumors in 100% of mice [21,22]. To alleviate animal suffering, all mice were euthanized by day 21 via carbon dioxide asphyxiation followed by cervical dislocation, as the majority of mice become moribund within days of this time point due to excessive tumor growth. Animals were routinely euthanized for experimental use between 9–11am, to minimize biologic variability due to light/dark animal housing cycles.

### Surface and intracellular staining for flow cytometry

At 7, 14, or 21 days after tumor challenge, tumor-bearing kidneys, tumor-free contralateral kidneys [22], spleens, livers, and adipose stromal cells (ASC) from pooled epididymal and renal fat pads were harvested and processed for flow cytometry [21,24]. Cells were stained and results acquired using multi-parameter flow cytometry on a BD LSR II (BD Biosciences) prior to analysis with FlowJo software (TreeStar Inc.). For MDSC: CD11c-APC/Cy7, CD11b-PE/Cy7, Ly6C-FITC, Ly6G-PErCP-Cy5.5, I-Ad-PE and Hoechst; in some experiments CCR2-PE or CCL2-PE or isotype controls were used. Antibodies were from BioLegend (San Diego, CA), or Millipore (Billerica, MA). Arginase I (PE conjugated anti-arginase I, R&D Systems, Minneapolis, MN) was detected via intracellular staining following surface staining as indicated above. Gates for arginase-positive events were based on fluorescence-minus one staining controls that lacked anti-arginase.

### MDSC enrichment and isolation

MDSC were harvested from multiple organs at the time points indicated. Briefly, organs were processed as described [21,24]. MDSC were enriched using Miltenyi anti-CD11b microbeads then sort-purified on a BD Aria II or Facs DiVa based on expression of the following markers after gating on live cells via Hoechst 33258 exclusion: Monocytic MDSC: CD45^+^/ CD11c^low^/ CD11b^+^/ Ly6C^+^Ly6G^low^; Granulocytic MDSC: CD45^+^/ CD11c^low^/ CD11b^+^/ Ly6G^+^/ Ly6C^dim^.

### T cell proliferation and inhibition assays

T cells were harvested from DUC18 TCR transgenic mice [25], incubated with CD8α microbeads (Miltenyi) and purified over two sequential MACS columns. The percentage of live DUC18 T cells was determined via flow cytometry based on surface co-expression of CD8α and Vβ8.3. T cells were used in proliferation assays [24] with sort-purified tumor-infiltrating MDSC or splenic MDSC from either NW or DIO tumor-bearing mice. MDSC inhibition of T cell proliferation was assessed by culturing naive DUC18 T cells (5 x 10^4^ cells per well) with tERK peptide-pulsed spDC [24,25] from control, tumor-free BALB/c mice (5x 10^3^ cells per well) and sort-purified tumor-infiltrating MDSC or splenic MDSC from tumor-bearing mice (5x 10^3^ cells per well). ^3H^Thymidine was added on d3 for the final 18 hr of culture. T cell inhibition was calculated as the % decrease in T cell proliferation with renal MDSC present relative to that seen for DUC18 T cells cultured with control, peptide-pulsed spDC alone [24].

### Cytokine and Chemokine evaluation by ELISA and BioPlex

Tumor homogenate samples were obtained from NW or DIO mice and frozen at-80C until use. Supernatant concentrations of the following cytokines and chemokines were determined via Multiplex analysis (Milliplex MAP kits, Millipore) on a BioRad BioPlex plate reader: IL-1β, IL-6, IL-9, TNFα, and MCP-1.

### Statistical Analyses

Statistical significance between experimental groups was determined by using paired or unpaired Student’s t-tests, with Welch’s corrections for unequal variances, as appropriate. (Prism, GraphPad Software Inc.). Throughout the manuscript, significance at p <. 05 is indicated by one asterisk, and p <. 01 is indicated by two asterisks.

## Results and Discussion

### Strain-specific baseline alterations in MDSC frequency in DIO mice

MDSC are well-described suppressors of protective anti-tumor immunity and accumulate in hosts in response to inflammation, trauma, and tumor-growth [13]. As obesity is associated with a chronic state of systemic inflammation [10], we asked whether baseline differences in cells with an MDSC phenotype were present in NW versus DIO mice. We analyzed tumor-free BALB/c mice after 20 weeks on either standard chow or high fat diet (HFD). At this time, DIO BALB/c mice exhibited several classic hallmarks of obesity: increased visceral fat as a percentage of total body weight, elevated serum leptin levels, and an inflammatory serum cytokine profile [21].

**Fig 1:**
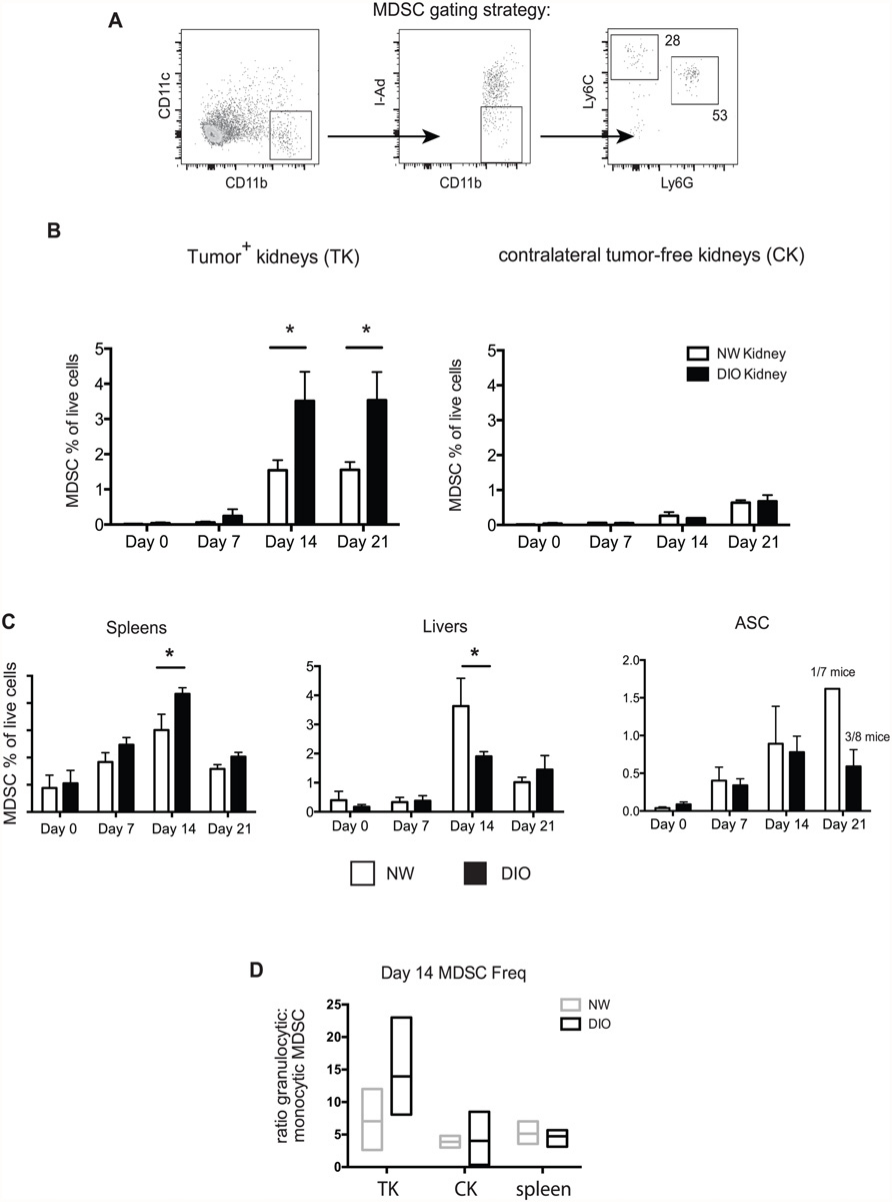
DIO mice have enhanced MDSC accumulation in renal tumors and spleens. (A) Flow cytometry gating strategy for MDSCs. (B) Frequencies of combined monocytic and granulocytic MDSC in tumor-bearing kidneys (TK) and tumor-free contralateral kidneys (CK) over time in DIO and NW mice. (C) Frequencies of combined monocytic and granulocytic MDSC in spleens, livers, and adipose stromal cells (ASC) from tumor-bearing mice in panel B. (D) Relative frequencies of granulocytic to monocytic MDSC are shown for the indicated organs at d14 post-tumor challenge. (B-D) For all, n = 4–8 mice/group, combined from three experiments. Bars indicate mean +/- s.d. Boxes indicate range with mean (horizontal bar). * = p < 0.05.

Our previous research had found functionally suppressive MDSC from tumor-bearing mice to be CD11c^neg^ [24], thus we gated on CD11c^neg^/ CD11b^+^/ I-A^d neg^/ Ly6C^+^ (monocytic) or CD11c^neg^/ CD11b^+^/ I-A^d neg^/ Ly6G^+^ (granulocytic) MDSC phenotype cells (Fig. 1A and S1 Fig.). We found no significant differences in the relative MDSC frequency (combined Ly6C^+^ and Ly6G^+^ subsets) or MDSC number in the kidney, spleen, liver, or epididymal/renal fat pad adipose stromal cell fraction (ASC) (S1 Fig.). Because this finding was in contrast to a prior report on MDSC accumulation in obese C57BL/6 mice [19], we examined MDSC frequencies in NW or DIO C57BL/6 mice after 8 weeks on diet, as Xia *et al*. had done. In doing so, we found increased percentages and numbers of MDSC in the spleens of these mice, in agreement with that report (S2 Fig.). However, we found no differences in the expression of CD115 or CD244.2, two proteins described as potential distinguishing markers of granulocytic MDSC versus neutrophils [26], on cells from DIO or NW C57BL/6 mice (S2 Fig.). Thus, in tumor-free mice, the onset of DIO is not universally accompanied by an accumulation of cells possessing an MDSC phenotype, and strain-specific differences exist.

### Obese mice have increased MDSCs in tumors and spleens

We next asked whether DIO led to enhanced MDSC accumulation in primary renal tumors, as this would contribute to both the immunotherapeutic failure we had observed previously in obese mice and the increased clinical prevalence of renal tumors in obese adults. NW or DIO BALB/c mice were challenged intra-renally on day 0 with Renca tumor cells to give rise to orthotopic kidney cancer, a process that we have shown leads to detectable renal tumors by day 7 and progressive tumor outgrowth in 100% of mice in the absence of therapy [22]. In this model, tumor outgrowth kinetics are minimally impacted by obesity [21].

The frequencies of combined Ly6C^+^ and Ly6G^+^ MDSC subsets were evaluated at days 7, 14, and 21. Tumors were not evaluated beyond day 21 due to rapid tumor growth and declining animal health [22]. We found increased percentages of MDSC in the tumor-bearing kidneys of both DIO and NW mice at days 14 and 21 post-tumor challenge, relative to respective day 0 controls (Fig. 1B). DIO mice had striking and significant elevations in MDSC percentages at these times, beyond what was observed in NW counterparts. Obesity-specific MDSC accumulation was not observed in tumor-free [22] contralateral kidneys. These results are in agreement with a recent report that found increased percentages of MDSC in renal tumors and spleens of obese versus lean mice at day 12 post-tumor challenge [20].

To determine if the combination of obesity and tumor growth led to alterations in MDSC accumulation at other sites, we examined spleens, livers, and epididymal/renal fat pad ASCs over the course of tumor outgrowth. In spleens, DIO mice again had a significant increase in MDSC frequency at day 14 relative to NW counterparts (Fig. 1C), in contrast to the liver, in which greater MDSC percentages were found in NW mice. Our examination of ASCs showed no obesity-related differences between days 0–14; a majority of mice had no detectable visceral adipose tissue remaining at day 21 (6 of 7 in NW mice and 5 of 8 in DIO mice) (Fig. 1C and data not shown).

As granulocytic (Ly6G^+^) and monocytic (Ly6C^+^) MDSC have been ascribed different functions *in vivo* [13,26,27], we next determined whether obesity caused a shift in MDSC subset composition. No statistical differences were observed in the granulocytic: monocytic MDSC ratios in tumor-bearing kidneys, contralateral kidneys, or spleens of DIO versus NW mice at day 21 (Fig. 1D), although there was a trend toward increased granulocytic MDSCs in the tumor-bearing kidneys of DIO mice. Thus, obesity was associated with elevated MDSC percentages only in primary tumors and spleens of mice with renal tumors and was not accompanied by significant shifts toward either the granulocytic or monocytic subtype.

### Obesity is associated with increased local expression of CCL2 in renal tumors

**Fig 2:**
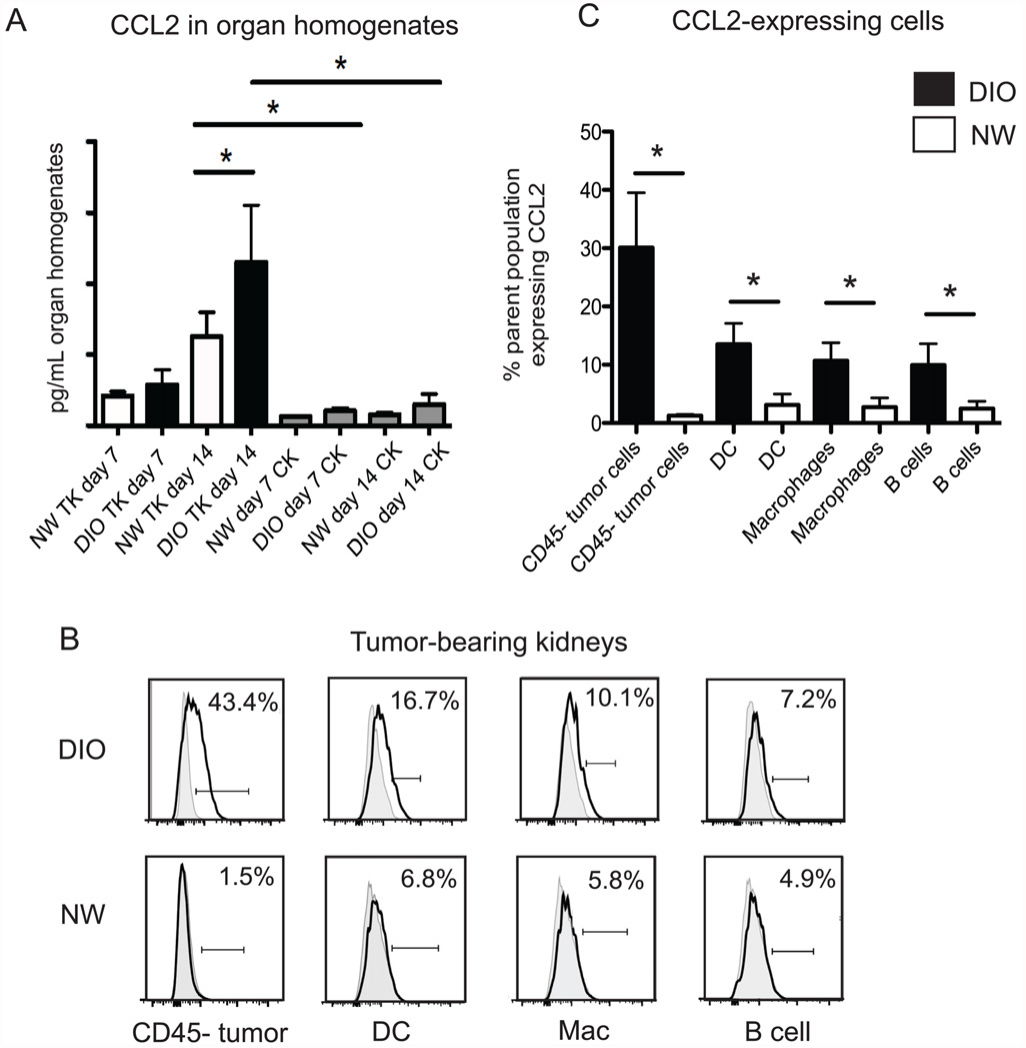
Obese mice have elevated concentrations of the MDSC chemoattractant CCL2 in renal tumors. (A) DIO and NW mice were challenged as in Fig. 1. Tumor-bearing kidneys (TK) or tumor-free contralateral kidneys (CK) were harvested at the times indicated. Homogenates were tested via Multiplex array for CCL2. n = 4–8 mice per group, combined from three experiments. Bars indicate mean +/- sd. * = p < 0.05. (B) Histograms showing intracellular CCL2 for the indicated cell populations. (C) Intracellular CCL2 from pooled DIO versus NW mice for the indicated cell populations, with n = 3–5 mice per group. Bars indicate mean +/- sd. * = p < 0.05.

Previous studies have shown that site-specific MDSC accumulation is regulated by cytokines and chemokines. In particular, CCL2 (monocyte chemoattractant protein-1, or MCP-1), TNFα, Interleukin-1β (IL-1β), and IL-6 have been identified as positive mediators of MDSC trafficking and accumulation [28,29]. Given the striking increase in MDSC prevalence in tumors from DIO mice between days 7 and 14, we examined tumor homogenates for the presence of each of the above factors at both time points in DIO and NW mice. Of these factors, only CCL2 showed a significant increase from DIO mice relative to NW mice at day 14 (Fig. 2A and S3 Fig.). As predicted by the lack of tumor growth [22] or MDSC accumulation in contralateral kidneys (Fig. 1), CCL2 concentrations remained low in contralateral kidneys from both DIO and NW mice throughout.

To determine whether obesity altered the cell types that produced CCL2 or changed the percentages of cells that secreted CCL2, we performed intracellular staining for CCL2 on cells from dissociated renal tumors from obese and NW mice at day 14. We found that tumors from obese mice had increased percentages of CD45^+^/CD11c^+^/MHC II^+^ DC, CD45^+^ /CD11b^+^/ MHC II^+^ macrophages, CD45^+^/B220^+^ B cells, and CD45^neg^ cells (non-leukocytes) that expressed CCL2 (Fig. 2B). Analysis of data pooled from multiple animals revealed that these differences were statistically significant (Fig. 2C). Thus, obesity led to heightened CCL2 secretion by multiple leukocyte populations and non-leukocyte cells within tumor masses, resulting in a local rise in CCL2 concentration within primary renal tumors.

Previous studies by our group and others have shown that CCL2 concentrations are elevated in the serum of RCC patients [18,30], and that CCL2 is produced at high levels by the tumor-associated macrophages that infiltrate human renal tumors [31]. Our murine results agree with these findings and suggest that additional cell populations should be examined in human renal tumors for their production of CCL2. Recently, CCL2 was found to promote breast cancer progression and angiogenesis through its effects on tumor-associated macrophages [32]. In this model, DIO C57BL/6 were found to express high levels of CCL2 in mammary glands, and transplantation of lean mice with humanized adipose stromal vascular fraction cells that over-expressed CCL2 identified novel adipocyte/ macrophage interactions mediated by CCL2/IL-1β/CXCL12 that promoted tumor progression through enhanced angiogenesis [32]. In our model, we found that obese mice had elevated CCL2, but not IL1β in renal tumors by day 7 after tumor transplantation (S3 Fig.). Therefore, it is possible that distinct CCL2-mediated axes promote tumor progression and/or angiogenesis in renal versus breast tumors.

In another recent study, adipocytes from DIO mice were found to secrete elevated levels of CCL2 and M-CSF after co-culture with melanoma cells [33]. These *in vitro* changes were thought to provide a mechanism for the increased accumulation of M2-phenotype macrophages seen in subcutaneous melanoma lesions of obese versus lean mice [33]. Our study complements these results by showing that renal tumors from obese mice also have elevated local CCL2, which is produced by a wide variety of cell types (dendritic cells, B cells, and macrophages) within the tumor microenvironment, possibly due to cross-talk between leukocytes and tumor cells. Collectively, findings from our lab and others demonstrate a link between suppressive myeloid cell accumulation in obese tumor-bearing mice and CCL2 production, suggesting that this is a common pathway that could be targeted therapeutically to improve immunotherapeutic efficacy in the obese.

Even in the absence of tumor growth, human obesity is known to be accompanied by elevated concentrations of CCL2 in the peripheral blood; pancreas; and subcutaneous, visceral, and omental adipose tissues [34–36]. High CCL2 has been shown to contribute to insulin resistance in the obese, as blocking signals through its receptor, CCR2, decreases weight gain and insulin resistance in DIO mice [37], and forced expression of CCL2 promotes insulin resistance [37,38]. In addition, CCL2 has been shown to contribute to macrophage infiltration into adipose tissue in genetically obese and DIO mice [37]. For these reasons, therapeutic strategies that neutralize CCL2 via antibody administration or block CCR2 signaling via small molecule inhibitors are being actively investigated (reviewed in [34]). At this time, however, neutralization of CCL2 has shown limited success [34], suggesting that targeting CCR2 may produce better therapeutic outcomes.

### Tumor-infiltrating MDSCs from obese mice do not have upregulated CCR2 expression

**Fig 3:**
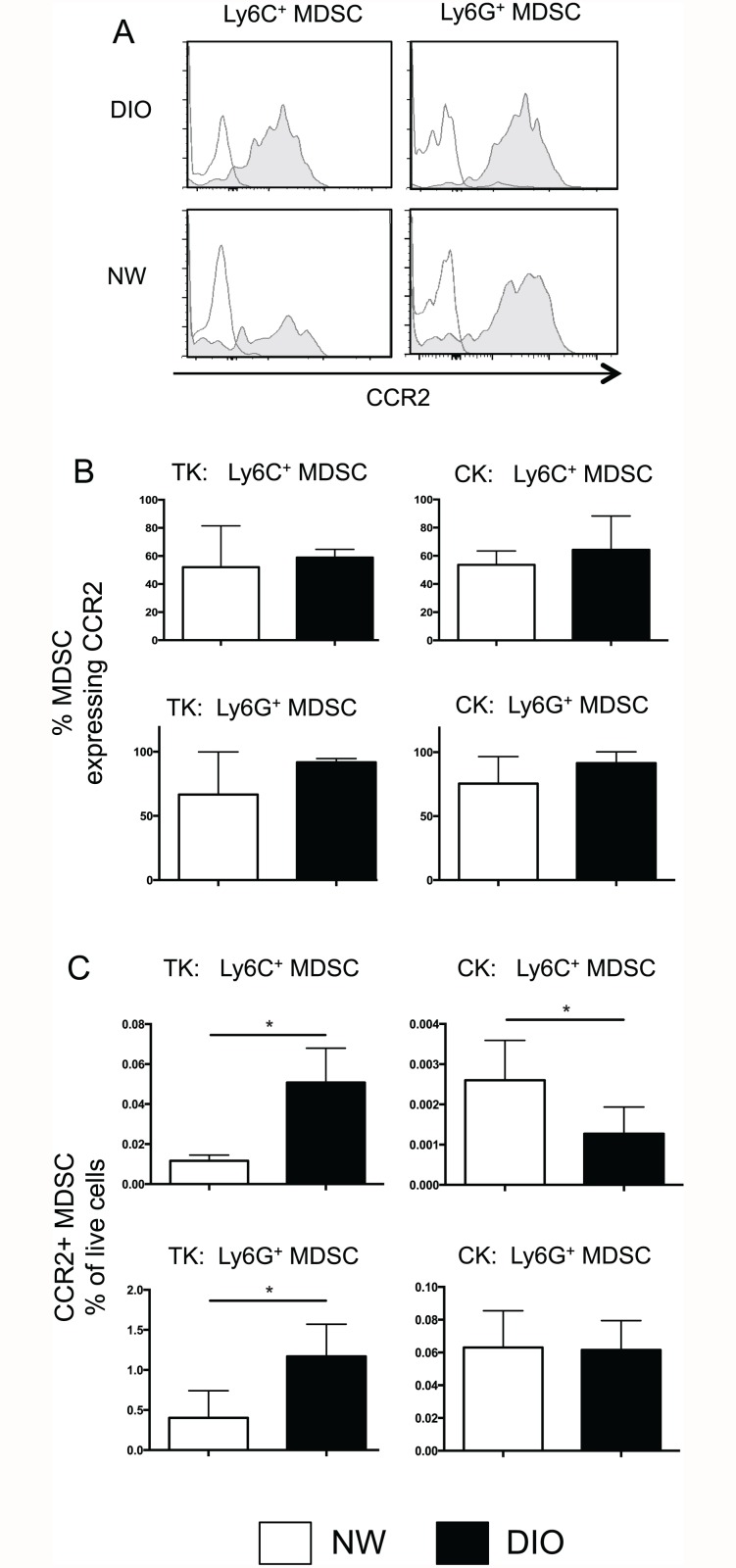
Equivalent surface expression of CCR2 on MDSCs from DIO and NW mice. (A) DIO and NW mice were challenged as in Fig. 1. Tumor-bearing kidneys (TK) or tumor-free contralateral kidneys (CK) were harvested at the times indicated. Histograms show representative surface expression of CCR2 on Ly6C^+^ or Ly6G^+^ MDSC from DIO or NW mice. Open histograms = isotype control; shaded histograms = CCR2 staining. (B) Pooled data showing the % of MDSCs that express CCR2 in tumor-bearing kidneys (TK) and contralateral kidneys (CK). Gating on Ly6C^+^ or Ly6G^+^ MDSC shows strong and equivalent expression of CCR2 on MDSC subpopulations from DIO and NW mice. (C) Increased overall frequencies of CCR2^+^ MDSC from both Ly6C^+^ and Ly6G^+^ subsets when calculated as a percentage of total live cells within tumor-bearing kidneys. For B and C, n = 4–6 mice per group from two experiments. Bars indicate mean +/- sd. * = p < 0.05.

The increased percentages of MDSCs in renal tumors of obese mice may result from up-regulation of the CCL2 receptor, CCR2, in addition to elevated local concentrations of CCL2 protein. We therefore examined CCR2 expression on MDSCs from tumor-bearing kidneys and tumor-free contralateral kidneys at d14 post-tumor challenge, as both CCL2 protein and MDSC accumulation were elevated at this time point (Fig. 2). We again gated on Ly6C^+^ monocytic or Ly6G^+^ granulocytic MDSC subpopulations (as in Fig. 1A) and examined surface CCR2 expression (Fig. 3). CCR2 protein was found on both subsets of MDSC from tumor-bearing kidneys in DIO and NW mice, with no change in the intensity of CCR2 staining (Fig. 3A). Equivalent percentages of monocytic and granulocytic MDSC expressed CCR2 in DIO and NW mice in both tumor-bearing and contralateral kidneys (Fig. 3B). However, when percentages of CCR2^+^ MDSCs as a fraction of the total live cells present were calculated, we again found significant increases in the overall frequency of both monocytic and granulocytic CCR2^+^ MDSC in tumor-bearing kidneys from DIO mice (Fig. 3C). Tumor-free contralateral kidneys contained few MDSCs (note scale change), and DIO mice had reduced percentages of CCR2^+^ monocytic MDSC as compared to NW counterparts (Fig. 3C). We conclude that the increased percentages of MDSCs within renal tumors from obese mice are due primarily to elevated local production of the myeloid chemoattractant CCL2, and not changes in expression of CCR2.

### Obesity does not alter the suppressive capacity of MDSC

**Fig 4:**
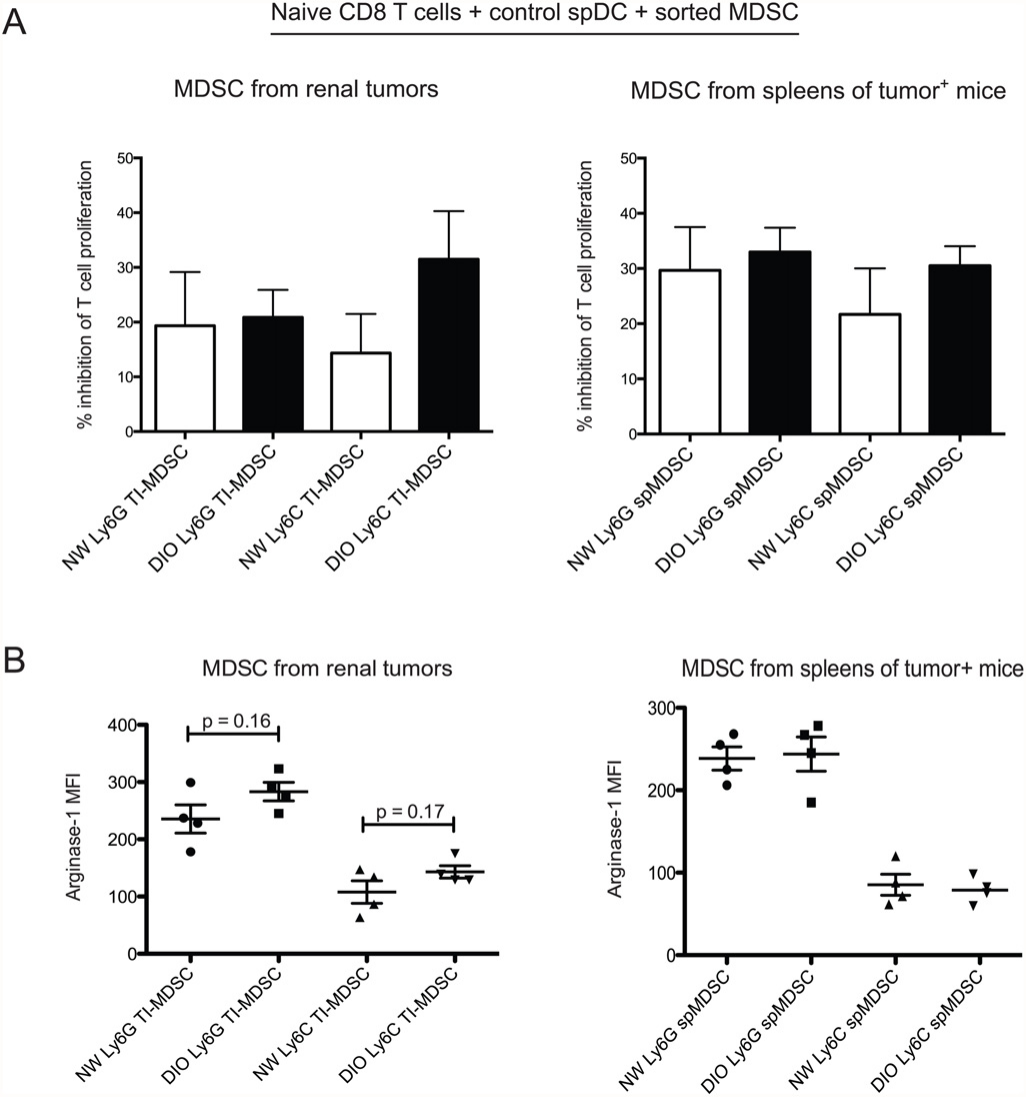
Obesity does not alter the suppressive capacity of MDSC. Tumor-bearing kidneys and spleens from the same mice were harvested between days 21–23. (A) Monocytic (Ly6C^+^) and granulocytic MDSC (Ly6G^+^) were sort-purified from each organ, then placed into culture with naive DUC18 T cells and stimulatory splenic DC from tumor-free NW mice. MDSC and stimulatory DC were pulsed with the DUC18 cognate peptide tERK. n = 6–8 mice per group, combined from three experiments. Bars indicate mean +/- sd. * = p < 0.05. TI = tumor-infiltrating: sp = splenic. (B) Bulk tumor and spleen homogenates were surface stained as described in Methods to distinguish monocytic versus granulocytic MDSC subpopulations, then stained intracellularly for arginase-1 expression. Dots indicate results from individual mice. No statistical differences were present in the percentages of arginase^+^ MDSC subpopulations from like organs in obese versus NW mice.

Finally, we asked whether MDSC from DIO mice had an altered suppressive capacity on a per cell basis, relative to MDSC from NW mice. DIO and NW mice were again challenged intra-renally on d0 with Renca cells, then tumors and spleens were harvested between days 21–24 when renal tumors were large and macroscopically visible. Monocytic and granulocytic MDSC subpopulations were sort-purified from both organs and examined for suppressive capacity *ex vivo*. Granulocytic tumor-infiltrating-MDSC (TI-MDSC) from DIO and NW mice showed nearly identical suppressive capacities *ex vivo* (Fig. 4A, left panel). Monocytic TI-MDSC from DIO mice showed a trend toward increased suppressive capacity, but this difference was not statistically significant. In addition, we found no differences in the per cell suppressive capacity of splenic Ly6C^+^ or Ly6G^+^ MDSCs from tumor-bearing DIO versus NW mice (Fig. 4A, right panel).

We then asked if obesity altered the intracellular expression of arginase, a protein known to mediate the suppressive functions of MDSC via depletion of L-arginine in the local tumor microenvironment [39]. L-arginine is conditionally required to support T cell proliferative bursts, and Rodriguez *et al*. had previously found that MDSC from RCC human subjects had high production of arginase that inhibited CD8 T cell expansion, CD3ς chain expression, and IFNγ production [40]. In agreement with our functional assessment of sort-purified MDSC, we found no differences in the intracellular expression of arginase between either granulocytic or monocytic TI-MDSC from obese versus lean mice (Fig. 4B). Although arginase expression was elevated in TI-MDSC subpopulations compared to splenic MDSC subpopulations, intracellular arginase expression was not altered by obesity. Thus, our results indicate that in this model of orthotopic renal cancer, obesity is associated with more robust immunosuppression in the tumor microenvironment, characterized by elevated CCL2 concentrations and increased percentages of functionally inhibitory MDSCs.

Antibody blockade of MCP-1/CCL2 is currently being tested for therapeutic efficacy in patients with metastatic prostate cancer. We and others have found that CCL2 is elevated in response to renal tumor growth in cancer patients [18,30,31]. Our results here suggest that CCL2 blockade, perhaps through small molecule inhibition of CCR2, may be particularly beneficial in obese patients, as it may normalize the percentages of immunosuppressive MDSC and permit immunostimulatory therapies to produce more robust antitumor immune responses.

Our previous work with this orthotopic murine renal tumor model demonstrated that obesity was associated with multiple defects in dendritic cell function: it decreased the ability of splenic dendritic cells to stimulate naive CD8^+^ T cell expansion, it led to increased frequencies of regulatory dendritic cells [24] within renal tumors, and it decreased IL-12 and TNFα production by dendritic cells in renal tumors [21]. These factors culminated in a decreased frequency of IFNγ^+^ effector CD8^+^ T cells within renal tumors from obese mice, and an inability of a T cell-dependent immunotherapy to induce tumor clearance, leading to fatal tumor outgrowth in obese mice. In contrast, following administration of the same immunotherapy, nearly 80% of lean mice cleared their renal tumors and experienced long-term survival [21].

Our results here add to the complex picture of immunologic changes that occur when obesity and tumor growth are present simultaneously. As no differences in the frequency of MDSC-phenotype cells were present prior to tumor challenge in any organ in obese versus lean BALB/c mice, we conclude that tumor-induced production of CCL2 is the main factor driving MDSC accumulation in obese tumor-bearing mice, rather than baseline, systemic inflammation present as a result of obesity. Although prior studies have demonstrated that CCL2 is elevated with obesity even in the absence of tumor growth [34,37], our results in both tumor-free DIO BALB/c and C57BL/6 mice suggest that these changes are insufficient to produce MDSC accumulation in healthy kidneys. In tumor-bearing mice, a high local concentration of CCL2, produced by a variety of cell types within the renal tumor microenvironment, appears to be needed for renal MDSC accumulation.

We determined here that obesity promotes MDSC accumulation within murine renal tumors and systemically in the spleen. Our cumulative results from this pre-clinical murine model suggest that obese renal tumor patients may have diminished responses to immune-stimulatory therapies. Given the fact that obesity is a major risk factor for RCC development and mortality [3,4,6,41], this possibility has clinical implications. Numerous prior studies have shown that human RCC is accompanied by increased local and systemic percentages of MDSCs [18,39,40,42], and that these changes are associated with worse clinical outcomes (reviewed in [42]). As granulocytic MDSCs are phenotypically similar to neutrophils, it is possible that prior human subject studies that found an increased neutrophil to leukocyte ratio as being an indicator of poor prognosis in RCC [43,44] were actually detecting increased frequencies of granulocytic MDSCs. In our current study, we found a trend toward increased granulocytic to monocytic MDSC frequency in renal tumors from obese mice, although this change did not reach statistical significance. We further found no change in the per-cell suppressive capacity or intracellular arginase expression in granulocytic MDSCs within renal tumors from obese versus lean mice (Fig. 4). However, as the overall percentages of total MDSC were increased in renal tumors and spleens from obese mice, the net result is stronger tumor-derived immunosuppression.

Based on our findings in this model of combined obesity and renal tumor growth, it is possible that blockade of CCL2/CCR2 or administration of known MDSC-depleting agents such as 5-fluorouracil [45] may prove beneficial as part of a combinatorial approach to immunotherapy in obese individuals with renal tumors. Although important biological differences exist between aggressively growing, transplanted murine renal tumors and human tumors that can take years to develop, our results are supported by a growing number of murine studies that have examined combined effects of obesity and tumor challenge on suppressive myeloid cell accumulation and function [20,32,33]. Thus, it appears that this trend is robust and broadly applicable to a variety of different tumor types. Collectively, murine results from our lab and others suggest a scenario in which obese cancer patients may suffer from more pronounced tumor-induced immune suppression, which could reduce the efficacy of administered anti-tumor immunotherapies. If true, this might necessitate tailoring immunotherapeutic approaches to cancer patients based upon their BMI status, a possibility that will need to be examined in future human subject studies.

## Supporting Information

S1 Fig Similar baseline frequencies and numbers of MDSC phenotype cells in DIO and NW BALB/c mice. 7–8 week old BALB/c mice were placed on either HFD or standard chow for 20 weeks. Obese mice were defined as described as in the Methods. Data are shown for cells with an MDSC phenotype: % (upper panels) and number (lower panels) based upon the combined prevalence of live CD11c^neg^/ CD11b^+^/ I-A^d neg^/ Ly6C^+^ (monocytic) and CD11c^neg^/ CD11b^+^/ I-A^d neg^/ Ly6G^+^ (granulocytic) cells. For all plots, n = 3–5 mice per group, combined from two independent experiments. Bars indicate mean +/- sd. No statistical differences were found between DIO and NW mice in any organ.
(EPS)

S2 Fig Elevated baseline frequencies and numbers of MDSC phenotype cells in spleens of DIO C57Bl/6 mice. 7–8 week old C57Bl/6 mice were placed on either HFD or standard chow for 8 weeks. Obese mice were defined as described as in the Methods. (A). The frequency (upper panels) and number (lower panels) of total cells with an MDSC phenotype are shown by organ. For all plots, n = 4–5 mice per group. Bars indicate mean +/- sd. (B) The expression of CD115 and CD244.2, both putative markers for differentiating granulocytic MDSC versus neutrophils, are shown for gated monocytic and granulocytic MDSC from DIO and NW spleens. Histograms are from one mouse, representative of 4–5 per group. * = p < 0.05.
(EPS)

S3 Fig Multiplex analysis of renal tumor homogenates shows no differences in IL-1β, TNFα, or IL-6 with obesity. DIO and NW mice were challenged as in Fig. 1. Tumor-bearing kidneys (TK) or tumor-free contralateral kidneys (CK) were harvested at the times indicated. Homogenates were tested via Multiplex array for the indicated cytokines. n = 4–8 mice per group, combined from three experiments. Bars indicate mean +/- sd. No significant differences were found between NW and DIO mice in TKs for any analyte tested.
(EPS)

## References

[1] Flegal KM, Carroll MD, Ogden CL, Curtin LR. Prevalence and trends in obesity among US adults 1999–2008. JAMA. 2010; 303: 235–241. 10.1001/jama.2009.2014 20071471

[2] Edwards BK, Noone AM, Mariotto AB, Simard EP, Boscoe FP, Henley SJ, et al Annual Report to the Nation on the status of cancer, 1975–2010, featuring prevalence of comorbidity and impact on survival among persons with lung, colorectal, breast, or prostate cancer. Cancer. 2014; 120(9): 1290–1314. 10.1002/cncr.28509 24343171 PMC3999205

[3] Chow WH, Dong LM, Devesa SS. Epidemiology and risk factors for kidney cancer. Nat Rev Urol. 2010; 7: 245–257. 10.1038/nrurol.2010.46 20448658 PMC3012455

[4] Wang F, Xu Y. Body mass index and risk of renal cell cancer: A dose-response meta-analysis of published cohort studies. Int J Cancer. 2014; 135(7): 1673–1686. 10.1002/ijc.28813 24615287

[5] Naya Y, Zenbutsu S, Araki K, Nakamura K, Kobayashi M, Kamijima S, et al Influence of visceral obesity on oncologic outcome in patients with renal cell carcinoma. Urol Int. 2010; 85: 30–36. 10.1159/000318988 20693825

[6] Colli JL, Busby JE, Amling CL. Renal cell carcinoma rates compared with health status and behavior in the United States. Urology. 2009; 73: 431–436. 10.1016/j.urology.2008.06.044 18701146

[7] Ladoire S, Bonnetain F, Gauthier M, Zanetta S, Petit JM, Guiu S, et al Visceral fat area as a new independent predictive factor of survival in patients with metastatic renal cell carcinoma treated with antiangiogenic agents. Oncologist. 2011; 16: 71–81. 10.1634/theoncologist.2011-S1-71 21212435 PMC3228050

[8] Steffens S, Grunwald V, Ringe KI, Seidel C, Eggers H, Schrader M, et al Does obesity influence the prognosis of metastatic renal cell carcinoma in patients treated with vascular endothelial growth factor-targeted therapy? Oncologist. 2011; 16: 1565–1571. 10.1634/theoncologist.2011-0213 22020210 PMC3233291

[9] Grivennikov SI, Greten FR, Karin M. Immunity, inflammation, and cancer. Cell. 2010; 140: 883–899. 10.1016/j.cell.2010.01.025 20303878 PMC2866629

[10] Gilbert CA, Slingerland JM. Cytokines, Obesity, and Cancer: New Insights on Mechanisms Linking Obesity to Cancer Risk and Progression. Annu Rev Med. 2012; 64: 45–57. 10.1146/annurev-med-121211-091527 23121183

[11] Park EJ, Lee JH, Yu GY, He G, Ali SR, Holzer RG, et al Dietary and genetic obesity promote liver inflammation and tumorigenesis by enhancing IL-6 and TNF expression. Cell. 2010;140: 197–208. 10.1016/j.cell.2009.12.052 20141834 PMC2836922

[12] Allavena P, Garlanda C, Borrello MG, Sica A, Mantovani A. Pathways connecting inflammation and cancer. Curr Opin Genet Dev. 2008; 18: 3–10. 10.1016/j.gde.2008.01.003 18325755

[13] Gabrilovich DI, Nagaraj S. Myeloid-derived suppressor cells as regulators of the immune system. Nat Rev Immunol. 2009; 9: 162–174. 10.1038/nri2506 19197294 PMC2828349

[14] Bunt SK, Sinha P, Clements VK, Leips J, Ostrand-Rosenberg S. Inflammation induces myeloid-derived suppressor cells that facilitate tumor progression. J Immunol. 2006; 176: 284–290. 16365420 10.4049/jimmunol.176.1.284

[15] Okwan-Duodu D, Umpierrez GE, Brawley OW, Diaz R. Obesity-driven inflammation and cancer risk: role of myeloid derived suppressor cells and alternately activated macrophages. Am J Cancer Res. 2013; 3: 21–33. 23359288 PMC3555202

[16] Ochoa AC, Zea AH, Hernandez C, Rodriguez PC. Arginase, prostaglandins, and myeloid-derived suppressor cells in renal cell carcinoma. Clin Cancer Res. 2007; 13:721s–726s. 17255300 10.1158/1078-0432.CCR-06-2197

[17] Walter S, Weinschenk T, Stenzl A, Zdrojowy R, Pluzanska A, Szczylik C, et al Multipeptide immune response to cancer vaccine IMA901 after single-dose cyclophosphamide associates with longer patient survival. Nat Med. 2012; 18: 1254–1261. 10.1038/nm.2883 22842478

[18] Wald G, Barnes KT, Bing MT, Kresowik TP, Tomanek-Chalkley A, Kucaba TA, et al Minimal changes in the systemic immune response after nephrectomy of localized renal masses. Urol Oncol. 2014; 32: 589–600. 10.1016/j.urolonc.2014.01.023 24768357 PMC4124886

[19] Xia S, Sha H, Yang L, Ji Y, Ostrand-Rosenberg S, Qi L. Gr-1+ CD11b+ myeloid-derived suppressor cells suppress inflammation and promote insulin sensitivity in obesity. J Biol Chem. 2011; 286: 23591–23599. 10.1074/jbc.M111.237123 21592961 PMC3123122

[20] James BR, Anderson KG, Brincks EL, Kucaba TA, Norian LA, Masopust D, et al CpG-mediated modulation of MDSC contributes to the efficacy of Ad5-TRAIL therapy against renal cell carcinoma. Cancer Immunol Immunother. 2014; 63: 1213–1227. 10.1007/s00262-014-1598-8 25143233 PMC4412276

[21] James BR, Tomanek-Chalkley A, Askeland EJ, Kucaba T, Griffith TS, Norian LA. Diet-Induced Obesity Alters Dendritic Cell Function in the Presence and Absence of Tumor Growth. J Immunol. 2012; 189: 1311–1321. 10.4049/jimmunol.1100587 22745381 PMC3401274

[22] Norian LA, Kresowik TP, Rosevear HM, James BR, Rosean TR, Lightfoot AJ, et al Eradication of metastatic renal cell carcinoma after adenovirus-encoded TNF-related apoptosis-inducing ligand (TRAIL)/CpG immunotherapy. PLoS ONE. 2012;7(2):e31085 10.1371/journal.pone.0031085 22312440 PMC3270031

[23] Sayers TJ, Wiltrout TA, McCormick K, Husted C, Wiltrout RH. Antitumor effects of alpha-interferon and gamma-interferon on a murine renal cancer (Renca) in vitro and in vivo. Cancer Res. 1990; 50: 5414–5420. 2117482

[24] Norian LA, Rodriguez PC, O’Mara LA, Zabaleta J, Ochoa AC, Cella M, et al Tumor-infiltrating regulatory dendritic cells inhibit CD8+ T cell function via L-arginine metabolism. Cancer Res. 2009; 69: 3086–3094. 10.1158/0008-5472.CAN-08-2826 19293186 PMC2848068

[25] Hanson HL, Donermeyer DL, Ikeda H, White JM, Shankaran V, Old LJ, et al Eradication of established tumors by CD8+ T cell adoptive immunotherapy. Immunity. 2000; 13: 265–276. 10981969 10.1016/s1074-7613(00)00026-1

[26] Youn JI, Collazo M, Shalova IN, Biswas SK, Gabrilovich DI. Characterization of the nature of granulocytic myeloid-derived suppressor cells in tumor-bearing mice. J Leukoc Biol. 2012; 91: 167–181. 10.1189/jlb.0311177 21954284 PMC3250305

[27] Schlecker E, Stojanovic A, Eisen C, Quack C, Falk CS, Umansky V, et al Tumor-Infiltrating Monocytic Myeloid-Derived Suppressor Cells Mediate CCR5-Dependent Recruitment of Regulatory T Cells Favoring Tumor Growth. J Immunol. 2012; 189: 5602–5611. 10.4049/jimmunol.1201018 23152559

[28] Tu S, Bhagat G, Cui G, Takaishi S, Kurt-Jones EA, Rickman B, et al Overexpression of interleukin-1beta induces gastric inflammation and cancer and mobilizes myeloid-derived suppressor cells in mice. Cancer Cell. 2008; 14: 408–419. 10.1016/j.ccr.2008.10.011 18977329 PMC2586894

[29] Huang B, Lei Z, Zhao J, Gong W, Liu J, Chen Z, et al CCL2/CCR2 pathway mediates recruitment of myeloid suppressor cells to cancers. Cancer Lett. 2007; 252: 86–92. 17257744 10.1016/j.canlet.2006.12.012

[30] Lukesova S, Kopecky O, Vroblova V, Hlavkova D, Andrys C, Moravek P, et al Determination of angiogenic factors in serum by protein array in patients with renal cell carcinoma. Folia Biol. 2008; 54: 134–140. 18808740 10.14712/fb2008054040134

[31] Daurkin I, Eruslanov E, Stoffs T, Perrin GQ, Algood C, Gilbert SM, et al Tumor-associated macrophages mediate immunosuppression in the renal cancer microenvironment by activating the 15-lipoxygenase-2 pathway. Cancer Res. 2011; 71: 6400–6409. 10.1158/0008-5472.CAN-11-1261 21900394

[32] Arendt LM, McCready J, Keller PJ, Baker DD, Naber SP, Seewaldt V, et al Obesity promotes breast cancer by CCL2-mediated macrophage recruitment and angiogenesis. Cancer Res. 2013; 73: 6080–6093. 10.1158/0008-5472.CAN-13-0926 23959857 PMC3824388

[33] Jung JI, Cho HJ, Jung YJ, Kwon SH, Her S, Choi SS, et al High-fat diet-induced obesity increases lymphangiogenesis and lymph node metastasis in the B16F10 melanoma allograft model: Roles of adipocytes and M2-macrophages. Int J Cancer. 2015; 136: 258–270. 10.1002/ijc.28983 24844408

[34] Panee J. Monocyte Chemoattractant Protein 1 (MCP-1) in obesity and diabetes. Cytokine. 2012; 60: 1–12. 10.1016/j.cyto.2012.06.018 22766373 PMC3437929

[35] Huber J, Kiefer FW, Zeyda M, Ludvik B, Silberhumer GR, Prager G, et al CC chemokine and CC chemokine receptor profiles in visceral and subcutaneous adipose tissue are altered in human obesity. J Clin Endocrinol Metab. 2008; 93: 3215–3221. 10.1210/jc.2007-2630 18492752

[36] Catalan V, Gomez-Ambrosi J, Ramirez B, Rotellar F, Pastor C, Silva C, et al Proinflammatory cytokines in obesity: impact of type 2 diabetes mellitus and gastric bypass. Obes Surg. 2007; 17: 1464–1474. 18219773 10.1007/s11695-008-9424-z

[37] Kanda H, Tateya S, Tamori Y, Kotani K, Hiasa K, Kitazawa R, et al MCP-1 contributes to macrophage infiltration into adipose tissue, insulin resistance, and hepatic steatosis in obesity. J Clin Invest. 2006; 116: 1494–1505. 16691291 10.1172/JCI26498 PMC1459069

[38] Tateya S, Tamori Y, Kawaguchi T, Kanda H, Kasuga M. An increase in the circulating concentration of monocyte chemoattractant protein-1 elicits systemic insulin resistance irrespective of adipose tissue inflammation in mice. Endocrinology. 2010; 151: 971–979. 10.1210/en.2009-0926 20056828

[39] Zea AH, Rodriguez PC, Atkins MB, Hernandez C, Signoretti S, Zabaleta J, et al Arginase-producing myeloid suppressor cells in renal cell carcinoma patients: a mechanism of tumor evasion. Cancer Res. 2005; 65: 3044–3048. 15833831 10.1158/0008-5472.CAN-04-4505

[40] Rodriguez PC, Ernstoff MS, Hernandez C, Atkins M, Zabaleta J, Sierra R, et al Arginase I-producing myeloid-derived suppressor cells in renal cell carcinoma are a subpopulation of activated granulocytes. Cancer Res. 2009; 69: 1553–1560. 10.1158/0008-5472.CAN-08-1921 19201693 PMC2900845

[41] Zhu Y, Wang HK, Zhang HL, Yao XD, Zhang SL, Dai B, et al Visceral obesity and risk of high grade disease in clinical t1a renal cell carcinoma. J Urol. 2013; 189: 447–453. 10.1016/j.juro.2012.09.030 23253956

[42] Chehval V, Norian LA. Effects of obesity on immune responses to renal tumors. Immunol Res. 2014; 59: 211–219. 10.1007/s12026-014-8533-0 24838144

[43] Jensen HK, Donskov F, Marcussen N, Nordsmark M, Lundbeck F, von der Maase H. Presence of intratumoral neutrophils is an independent prognostic factor in localized renal cell carcinoma. J Clin Oncol. 2009; 27: 4709–4717. 10.1200/JCO.2008.18.9498 19720929

[44] Sejima T, Iwamoto H, Morizane S, Hinata N, Yao A, Isoyama T, et al The significant immunological characteristics of peripheral blood neutrophil-to-lymphocyte ratio and Fas ligand expression incidence in nephrectomized tumor in late recurrence from renal cell carcinoma. Urol Oncol. 2011; 31(7): 1343–1349. 10.1016/j.urolonc.2011.09.008 22153754

[45] Vincent J, Mignot G, Chalmin F, Ladoire S, Bruchard M, Chevriaux A, et al 5-Fluorouracil selectively kills tumor-associated myeloid-derived suppressor cells resulting in enhanced T cell-dependent antitumor immunity. Cancer Res. 2010; 70: 3052–3061. 10.1158/0008-5472.CAN-09-3690 20388795

